# Crystalline modification of a rare earth nucleating agent for isotactic polypropylene based on its self-assembly

**DOI:** 10.1098/rsos.180247

**Published:** 2018-05-16

**Authors:** Yuanming Zhang, Tingting Sun, Wei Jiang, Guangting Han

**Affiliations:** 1 College of Textiles, Donghua University, Shanghai 200051, People's Republic of China; 2 Laboratory of New Fibre Materials and Modern Textile, The Growing Base for State Key Laboratory, Qingdao University, Qingdao 266071, People's Republic of China

**Keywords:** crystalline, nucleating, polypropylene, self-assembly

## Abstract

In this paper, the crystalline modification of a rare earth nucleating agent (WBG) for isotactic polypropylene (PP) based on its supramolecular self-assembly was investigated by differential scanning calorimetry, wide-angle X-ray diffraction and polarized optical microscopy. In addition, the relationship between the self-assembly structure of the nucleating agent and the crystalline structure, as well as the possible reason for the self-assembly behaviour, was further studied. The structure evolution of WBG showed that the self-assembly structure changed from a needle-like structure to a dendritic structure with increase in the content of WBG. When the content of WBG exceeded a critical value (0.4 wt%), it self-assembled into a strip structure. This revealed that the structure evolution of WBG contributed to the *K*_β_ and the crystallization morphology of PP with different content of WBG. In addition, further studies implied that the behaviour of self-assembly was a liquid–solid transformation of WBG, followed by a liquid–liquid phase separation of molten isotactic PP and WBG. The formation of the self-assembly structure was based on the free molecules by hydrogen bond dissociation while being heated, followed by aggregation into another structure by hydrogen bond association while being cooled. Furthermore, self-assembly behaviour depends largely on the interaction between WBG themselves.

## 1. Introduction

In the last decade, isotactic polypropylene (PP) has attracted common attention owing to its favourable price/performance ratio and many possible modifications. PP is a polymer with multiple crystalline phases [1,2]: α-, β- and γ-phases. Only the α- and β-phases possess application relevance. Normally, PP crystallizes into an α-crystal, which has low toughness, while the β-phase shows excellent toughness as well as thermal performance [3–5]. The formation of a β-crystal needs special conditions: temperature gradient [6], quenching [1], shearing [7–9] and adding β-nucleating agents [10–12]. Among these methods, adding β-nucleating agents is considered to be the most efficient one.

The development of different kinds of β-nucleating agents has raised lots of concerns. Recently, Feng *et al.* [13] have proved that some complexes of rare earth metals possess β-nucleating ability. Based on the above discovery, a novel rare nucleating agent—WBG—was synthesized. According to some patents and papers [14,15], this novel nucleating agent might be a complex composed of lanthanum and some specific ligands [16]. Moreover, WBG has been proved to be a high-performance β-nucleating agent, which could induce about 90% β-crystal with a content of just 0.3 wt%. The mechanical behaviour and non-isothermal crystallization behaviour of PP with WBG has been widely studied for decades [17–19] in pioneering works.

It has been proved that crystallization structure has an important effect on the final properties. Crystallization includes two stages: nucleation and crystal growth. The former stage is related to the topological structure of the nucleating agent, which determines the final crystallization structure. However, little attention was paid to the structure evolution of WBG in the PP matrix. In pioneering works, some other β-nucleating agents have been proved to self-assemble into various topological structures according to the final temperature of heating and the content of β-nucleating agents [20–22]. Varga and Menyhárd studied a commonly used nucleating agent—*N*,*N*′-dicyclohexyl-2,6-naphthalenedicarboxamide (NJS)—and found that this nucleating agent could self-assemble into a needle-like structure at low nucleating agent concentration and into a dendritic structure at a larger concentration when being heated at a high temperature. It is not hard to understand that topological structures of β- nucleating agents are related to their composition, and various nucleating agents self-assemble into different structures. While the self-assembly behaviour of WBG is rarely studied, this paper aims to study its self-assembly behaviour and crystalline modification.

In this paper, a kind of rare earth nucleating agent—WBG—was introduced into PP, and the self-assembly behaviour, the crystalline modification of the rare earth nucleating agent for PP and the relationship between the topological structure of the nucleating agent and the crystalline structure were investigated; also, the possible reason for the self-assembly behaviour was further studied.

## 2. Experimental section

### 2.1. Materials and reagents

Commercially available isotactic PP (F401, supplied by Yangzi Petrochemical Co. Ltd., PR China) was used as the basic material throughout the study. The material was characterized by a melt flow index of 2.5 g 10 min^−1^ (230°C, 2.160 N), an isotacticity index of 96.5% and a weight-average molecular weight of approximately 110 000. The specific β-nucleating agent used throughout this work was the rare earth β-nucleating agent (WBG, supplied by Winner Functional Materials Co. Ltd., PR China).

### 2.2. β-Nucleated polypropylene preparation

To obtain homogeneous distribution of the WBG in the PP matrix, master batches were prepared by premixing PP with 1 wt% of WBG nucleating agent in a twin-screw extruder (SHJ-20). The master batches were then mixed with PP again in the twin-screw extruder. The resulting concentration of the WBG nucleating agent was 0.1–0.5 wt%, at a step of 0.1 wt%. Subsequently, the melt from the extruder was drawn into a water bath (with a final radius of 2.5 mm). The temperature of the water bath was set by a temperature-controlling device, and the temperature was set to 20°C. Detailed parameters of the above process are summarized in table 1.

**Table 1.** Process parameters of β-nucleated polypropylene.

temperature zone	1	2	3	4
temperature setting	190°C	200°C	210°C	220°C
other parameters	twin-screw speed	cooling temperature	cooling time	draw ratio
	60 r.p.m.	20°C	1200 s	1

### 2.3. Wide-angle X-ray diffraction

PP with different content of WBG was carefully cut into small fragments with a razor blade. Subsequently, the fragments were selected with a 360 hole sieve. A Bruker of D8 (Bruker, ADVANCE) diffractometer using Cu K*α* radiation was used for the wide-angle X-ray diffraction (WAXD) analysis of all studied materials. The diffractometer measured the radial diffracting range: 2*θ* = 1.5°–30° in transmission, by an area detection system, LynxEye. The diameter of the detector pinhole was 0.5 mm.

The relative content of the β-phase was evaluated for PP with different content of WBG. The relative content of the β-phase (*K*_β_) was calculated by the method of Jones *et al.* [1]:

2.1 $$K_{\beta }=\frac{I_{\beta (300\text{) }}}{I_{\alpha (110\text{) }}+I_{\alpha (040\text{) }}+I_{\alpha (130\text{) }}+I_{\beta (300\text{) }}},$$

where *I*_β(300)_ is the integral intensity of (300) diffraction of the β-phase, and *I*_α(110)_, *I*_α(040)_ and *I*_α(130)_ are the integral intensities of (110), (040) and (130) diffraction, respectively.

### 2.4. Differential scanning calorimetry

The result of differential scanning calorimetry (DSC) is employed to analyse the thermal properties of β-nucleated PP, including the melting behaviour and crystallization behaviour. The DSC tests were performed with a Linseis Stsa differential scanning calorimeter at a heating rate of 5°C min^−1^ and with a nitrogen flow of 20 ml min^−1^, and the crystallization behaviour was investigated at a cooling rate of 5°C min^−1^. The relative content of the β-crystal was calculated according to the following equation:

2.2 $$\beta_{c}=\frac{X_{\beta }}{X_{\beta }+X_{\alpha }}=\frac{\Delta H_{\beta }/\Delta H_{\beta^{0}}}{\left(\Delta H_{\beta }/\Delta H_{\beta^{0}})+(\Delta H_{\alpha }/\Delta H_{\alpha^{0}}\right)},$$

where $\Delta H_{\beta }$ is the fusion heat caused by the β-crystal in the sample and $\Delta H_{\beta^{0}}$ is the standard fusion heat of β-crystal PP; similarly, $\Delta H_{\alpha }$ is the fusion heat which is caused by the α-crystal in the sample and $\Delta H_{\alpha^{0}}$ is the standard fusion heat of α-crystal PP.

### 2.5. Polarized optical microscope

The spherulitic morphologies of PP and PP with different content of WBG, as well as the structure evolution of WBG, were observed with an ECLiPSE E600POL polarized optical microscope (POM) with a hot stage. The hot stage could control the terminal temperature and the cooling rate. The samples were prepared on a heating plate by melting and squeezing into films. Each film was heated to 220°C and then cooled to room temperature at a cooling rate of 5°C min^−1^. The non-isothermal crystallization process was observed under the POM.

### 2.6. Fourier transform infrared spectrum

The Fourier transform infrared (FTIR) spectrum was employed to analyse the mechanism of self-assembly behaviour from the view of bond action. PP was pressed into a tablet during the melting process. The sample was scanned from 4000 cm^−1^ to 500 cm^−1^ 16 times.

## 3. Result and discussion

### 3.1. The self-assembly structure of WBG and the analysis for mechanism

**Figure 1.**
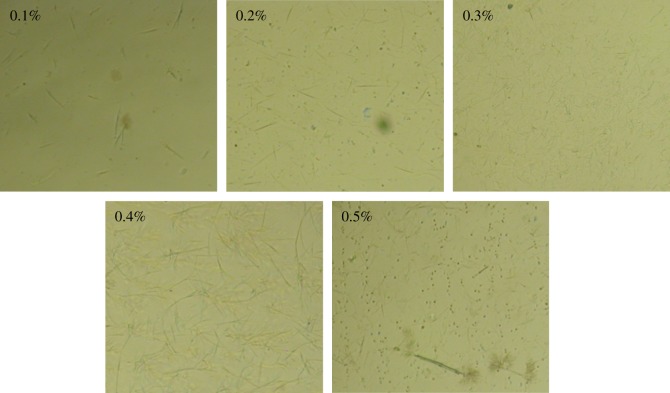
The self-assembled structure of WBG with different content.

It is proverbial that macromolecular crystallization includes two stages: nucleation and crystal growth. The former stage is related to the topological structure of the nucleus, which determines the final crystallization structure. Thus, the self-assembly of WBG was studied first. It was found that the topological structure of WBG is related to its content. When adding 0.1% WBG, the formation of self-assemblies could been seen at about 140°C, and this temperature increases to about 150°C in the case of 0.5% WBG. In addition, the morphology of aggregation versus the different content of WBG is shown in figure 1. It can be seen that WBG self-assembles into a needle-like structure when its content is 0.1% and 0.2%. In the case of 0.3 wt% and 0.4 wt%, WBG self-assembles into dendritic structure, and the size of the dendritic structure with 0.4 wt% content is larger. When the content reaches 0.5 wt%, it is interesting to find a strip structure. With the increase in content, the topological structure changes.

**Figure 2.**
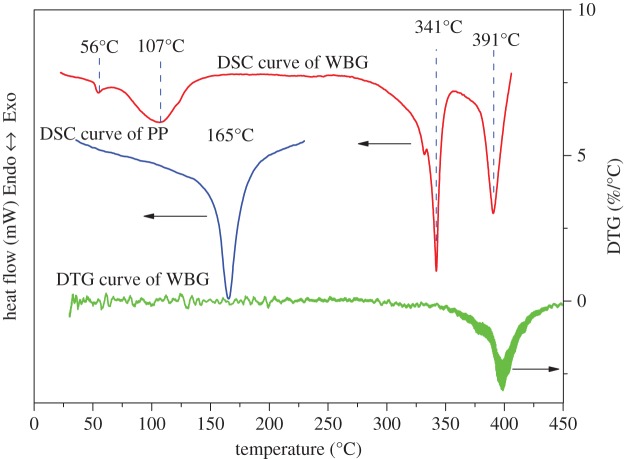
The melting behaviours of WBG and pure PP.

**Figure 3.**
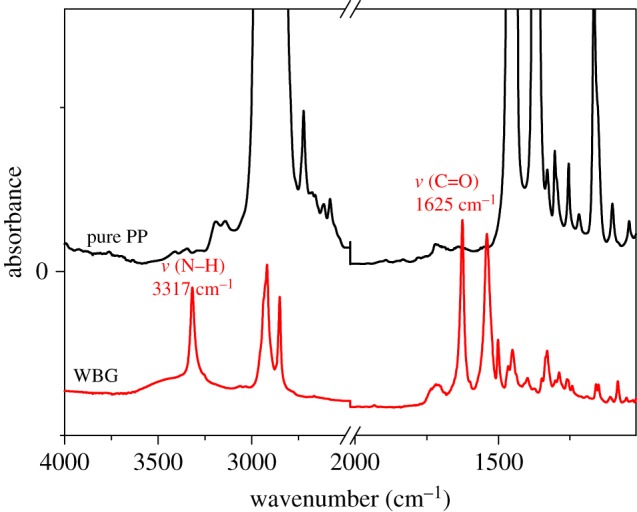
The FTIR spectra of pure PP and WBG.

**Figure 4.**
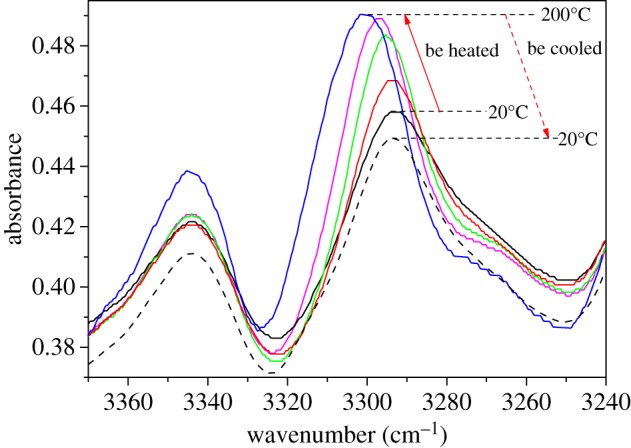
The change of N–H while being heated and after being cooled.

**Figure 5.**
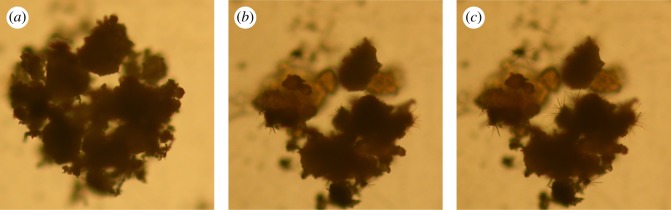
The morphology of WBG when heated to 220°C and then cooled from 220°C to 20°C: (*a*) original morphology of agglomerates; (*b*) needle-like structure emerging while being heated to 220°C; and (*c*) needle-like structure enhances when being cooled.

It is accepted that self-assembled structure of the β-nucleating agent is related with its solubility in the polymer melt [23,24]. According to the thermodynamic analyses of WBG and PP (figure 2), we found that when WBG is heated from room temperature to 450°C, two endothermic peaks of WBG appear at 56°C and 107°C, respectively. In combination with the differential thermal gravity (DTG) curve of WBG, there is no quality change of WBG at 56°C and 107°C, thus the two endothermic peaks are melting peaks. Actually, the melting temperature of stearic acid is 56–69°C [25,26], and that of lanthanum stearate is 100–110°C [27]. It is not difficult to understand that the two endothermic peaks are attributable to the melting behaviour of organic ligands and inorganic substances inside WBG, respectively. In addition, we can find another endothermic peak with no quality change at 341°C; this is also a melting peak. The decomposition temperature is 391°C. While moving on to pure PP, it can be seen that the melting temperature of pure PP is 165°C. The above results indicate that the self-assembly of WBG occurs based on its partial dissolution in the PP matrix. The self-assembly behaviour is a liquid–solid transformation of WBG followed by a liquid–liquid phase separation of molten isotactic PP and WBG. The dibenzylidene sorbitol derivatives (another nucleating agent) also has the same behaviours [28]. The reason that causes the self-assembly behaviour (a liquid–solid transformation of WBG followed by a liquid–liquid phase separation of molten isotactic PP and WBG) should be discussed. We surmise the reason may include two aspects: one is the interaction between the PP matrix and WBG; the other is the interaction between WBG themselves. To reveal the reason, the FTIR spectra in figure 3 was studied. From figure 3, it can be seen that pure PP has a typical absorbance band of C–H groups at about 2900 cm^−1^, while WBG has three typical absorbance bands of the N–H, C–H and C=O groups, which appear at about 3317 cm^−1^, 2900 cm^−1^and 1625 cm^−1^, respectively. The absorbance bands of C–H groups of isotactic PP and WBG overlap, while the absorbance band of N–H is unique to WBG. So we studied the change of N–H while being heated and after being cooled. Figure 4 shows that the band of N–H shifts to a higher frequency while being heated to 220°C, and it is a blue shift [29–31]. While being cooled to room temperature, the band of N–H shifts to a lower frequency; it is a red shift. It is reported [32–34] that a blue shift is owing to the weakening of a hydrogen bond or dissociation; correspondingly, the red shift is attributed to the strengthening of a hydrogen bond or association. This supports the fact that when being heated the WBG turns into free molecules by hydrogen bond dissociation, and when being cooled, the free molecules aggregate into another structure by hydrogen bond association. This implies that the self-assembly behaviour has a strong relationship with the interaction between WBG themselves. With regard to the second aspect, it is difficult to study the effect of interaction between the PP matrix and WBG on self-assembly behaviour. However, we heated WBG from room temperature to 220°C and then cooled it to room temperature. It can be found from figure 5 that while being heated to 220°C, needle-like structures appear (figure 5*b*). In addition, the size of it enhances with reduction in temperature. This implies that the self-assembly behaviour of WBG can occur without dissolution in the PP matrix. This supports the fact that the self-assembly behaviour of WBG may have a weak relationship with the interaction between the PP matrix and WBG. This aspect should be further studied.

### 3.2. The effect of WBG on the crystallization morphology of polypropylene

**Figure 6.**
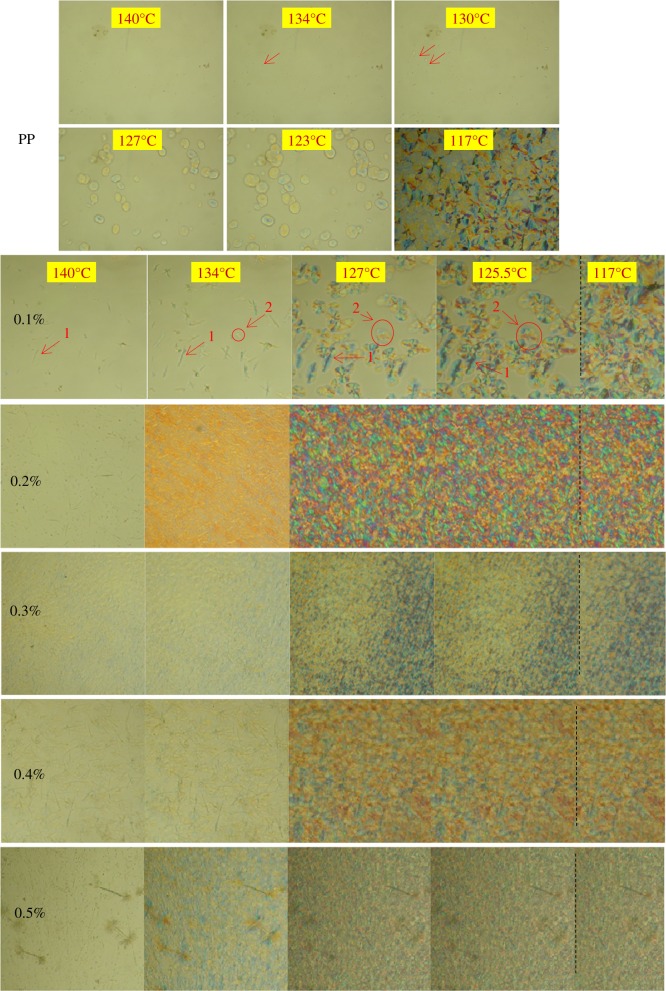
POM photos of WBG-modified PP samples with different content of WBG during the cooling process.

The crystallization morphology of PP with different β-nucleating agent content is monitored by POM and it is shown in figure 6. It is obvious that in the case of no additives, nuclei emerges at a lower temperature of about 134°C and causes the formation of an *α*-crystal (The morphology of spherulites is shown in figure 1). Furthermore, the growth of spherulites is a process of homogeneous nucleation. The crystal grows slowly and the complete crystallization morphology forms at near 117°C. When 0.1 wt% WBG is added into the PP matrix, as mentioned above WBG can self-assemble into needle-like structures, which induces crystal growth. Heterogeneous nucleation occurs all over the crystallization process. It also can be seen that the needle-like structure (position 1) appears at 140°C. The crystal nuclei appear earlier than pure PP. To explain this result, recall figure 5, which shows that the self-assembly behaviour of WBG can occur while being heated to 220°C, and this may be attributed to the early appearance of heterogeneous nuclei during the cooling process. In addition, it is interesting to find that another type of nuclei (position 2) appears in the matrix when the temperature reaches 134°C. The time of emergence and the morphology of these nuclei (position 2) are similar with those in pure PP. From the view of the morphology of crystals formed in position 2 at 125.5°C, it is supported that the crystal formed in position 2 in an *α*-crystal. This supports the fact that *α*-nuclei and complete *α*-spherulites can form in PP with a low content of WBG. Note again the crystal growth in position 1; an obvious transcrystalline structure forms at 125.5°C. (The morphology of transcrystalline structure is shown in figure 1.) The transcrystalline structure developing around the needles has been studied by Varga *et al.* [35], and the *α*-crystal and the *β*-crystal have been proved to form in different positions around the needle. This means that the transcrystalline structure is the combination of both the *α*- and the *β*-crystal. When the content of WBG increases from 0.2 to 0.5 wt%, it is obviously shown that the complete crystallization morphology forms at near 127°C, which signifies a faster spherulite growth rate. This is also attributed to the self-assembly structure of WBG. The *β*-crystal mainly grew from the tip of needles. When the topological structure changed into a dendritic structure with increasing content of WBG, the active point for *β*-crystal growth increased, which would lead to a faster growth rate. Finally, a snowflakes-like crystal could be found based on the dendritic topological structure. Notably, the strip structure can be observed when the crystallization process is complete. Furthermore, it can be seen from the crystallization process that PP crystallizes just around its two tips and the strip structure is unfavourable to crystal growth. Accordingly, the self-assembly behaviour is crucial to crystallization morphology.

### 3.3. The effect of WBG on the thermal properties and crystallization structure of polypropylene

**Figure 7.**
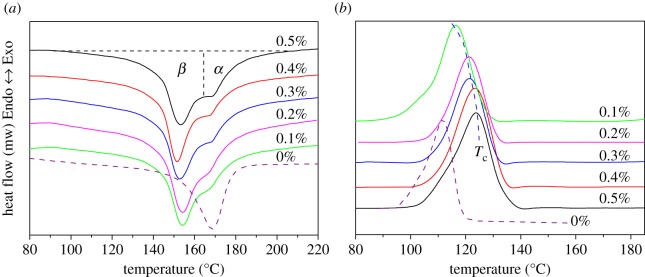
(*a*) Effect of the content of WBG on the melting behaviours and (*b*) crystallization curves of β-nucleated PP.

Figure 7 shows the effects of the content of WBG on the melting and crystallization behaviours of PP. From figure 7*a*, it can be seen that pure PP only has one melting peak at a temperature of about 168°C, while WBG-modified PP exhibited two melting peaks at temperatures of about 153°C and 168°C, which correspond to the melting temperature of the β- and α-crystal. It can be observed that the peak area of the β-crystal is bigger than that of the α-crystal, an indication of high relative content of the β-crystal. The relative content of the β-crystal (*β*_c_) is calculated based on the melting curve displayed in table 2. It can be seen that *β*_c_ increases first and then decreases with increase in the content of WBG, and samples are rich in the β-crystal if the content of WBG reaches 0.4 wt%. Figure 1*b* shows the crystallization curve of PP with different content of WBG. This implies that the crystallization peak temperature (*T*_c_) of pure PP is around 111.6°C. The addition of WBG produces a marked shift of *T*_c_ towards a higher temperature. In addition, *T*_c_ shifts to a higher temperature with the increase in the content of WBG. The values of *T*_c_ are given in table 2. The shift of the crystallization peak temperature is from 116.4°C in 0.1 wt% to 123.8°C in 0.5 wt%. The shift in *T*_c_ is consistent with that of the formation temperature of complete crystallization morphology. The shift is related to the self-assembly structure of WBG. From a kinetic viewpoint, Xiao *et al.* [36] studied the fold surface free energy *δ*_e_ using the Lauritzen–Hoffman theory [37], which revealed that the addition of WBG can decrease fold surface free energy. It is well accepted that the smaller the fold free energy of the crystallization surface, the easier it is for the macromolecule chain to form a crystal structure. Thus, high temperature is enough to induce crystallization behaviour, and the effect is more remarkable with increase in the content of WBG.

**Table 2.** The value of *β*_c_, *K*_β_, *T*_c_ of PP with different content of WBG.

content of WBG	*β*_c_ (%)	*K*_β_ (%)	*T*_c_ (°C)
0	0.00	0.00	111.6
0.1	70.19	51.62	116.4
0.2	72.09	90.04	120.6
0.3	72.75	92.08	121.6
0.4	73.96	92.96	123.4
0.5	71.73	84.26	123.8

**Figure 8.**
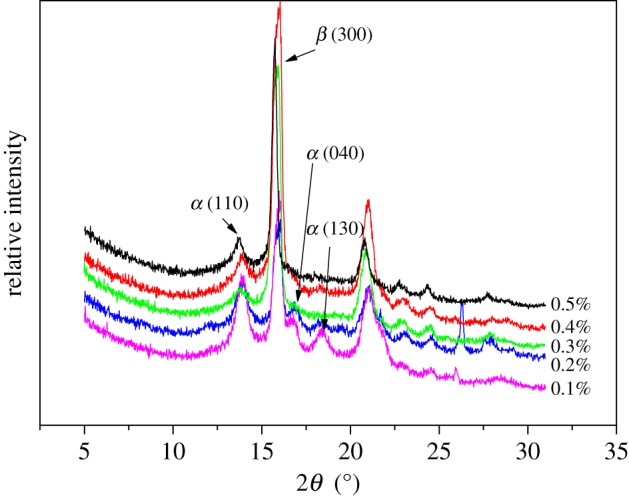
WAXD patterns of PP with different content of WBG.

The data of WAXD of PP with different contents of WBG are shown in figure 8. It shows that *α*(110), *α*(040) and *α*(130) crystal plane diffraction can be detected in PP with 0.1 wt%; the diffraction intensities of the *α*(110), *α*(040) and *α*(130) crystal plane decreases with increase in the content of WBG. Furthermore, when the content reaches 0.4 wt%, the *α*(040) and *α*(130) crystal plane diffraction vanishes. The relative contents of the β-crystal calculated from the results of WAXD are displayed in table 2. From table 2, it can be seen that the value of *K*_β_ increases first and then decreases with increase in the content of WBG. The relative content of the β-crystal can reach 92% in 0.4 wt%, which is the critical content of WBG. Actually, there are various critical contents of different nucleating agents to get products rich in β-nucleated PP [38–40]. There was some connection between the content of WBG and the topological structure, as well as the β-crystal content. With increase in WBG content, more macromolecules of the nucleating agent participate in the self-assembly behaviour. Consequently, the hydrogen bond association was strengthened. More macromolecules were aggregated, which led to a topological structure with a larger size. As we can see, the topological structure changed from a needle-like structure to dendritic structure. The dendritic structure had more active points, which would induce more crystal growth of PP on its surface. The results of both DSC and WAXD indicate a reduction in β-crystal content when adding 0.5 wt% WBG. Recalling the final crystallization morphology in figure 6, as can be seen, the self-assembly structure that formed with addition of 0.5 wt% WBG is unfavourable to crystal growth. That reduces the nucleation efficiency of WBG. In the analysis for the mechanism of self-assembly behaviour, the topological structure changes from a needle-like structure to dendritic structure with the increase in the content of WBG. That leads to an increase of β-crystal content. However, when the content of WBG exceeds a critical value, the interaction between WBG themselves enhances, which is attributed to the appearance of a strip structure. This aggregation structure can reduce the nucleation efficiency of WBG.

## 4. Conclusion

This paper focuses on the crystalline modification of a rare earth nucleating agent for PP, the self-assembly behaviour, as well as the relationship between the topological structure of the nucleating agent and the crystalline structure. The experimental results showed that the self-assembly structure changed from a needle-like structure to dendritic structure with increase in the content of WBG. When the content of WBG exceeded a critical value (0.4 wt%), it self-assembled into a strip structure. The relative content of the β-crystal increased first and then decreased with increase in the content of the nucleating agent; the content of 0.4 wt% could get products rich in β-nucleated PP. The crystallization morphology also depends on the content of the nucleating agent. Further study shows that the self-assembly structure was affected by interaction between WBG themselves. When the content of WBG exceeds a critical value, the interaction enhances. That is attributed to the appearance of a strip structure. Furthermore, this aggregation structure can affect the content of the β-crystal and the crystallization morphology owing to the reducing efficiency of WBG.

## Supplementary Material

Figure S1

## Data accessibility

This article does not contain any additional data.
