# Supplementary material for: Crystalline modification of a rare earth nucleating agent for isotactic polypropylene based on its self-assembly
Source: R Soc Open Sci. 2018 May 16;5(5):180247. doi: 10.1098/rsos.180247 (PMC5990779; doi:10.1098/rsos.180247)
Supplement: Figure S1 [file rsos180247supp1.docx]

**Crystalline modification of rare earth nucleating agent for isotactic polypropylene based on its self-assembly**

Yuanming Zhang^a,b^, Tingting Sun^a^, Wei Jiang^b^,Guangting Han^a,b,^[[1]](#footnote-2)^*^,

*^a^ College of Textiles, Donghua University, Shanghai, 200051, P.R. China*

*^b^ Laboratory of New Fibre Materials and Modern Textile, the Growing Base for State Key Laboratory, Qingdao University, Qingdao266071, P.R. China*

Keywords: crystalline, nucleating, polypropylene, self-assemble


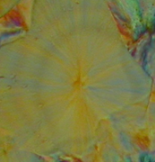

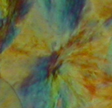

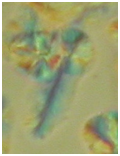


Figure S1. The morphology of spherulites at high magnification. (a) and (b) shows the spherulites morphologys of pure PP. (c) shows the transcrystalline structure of PP with 0.1% WBG.

a

b

c

1. *Corresponding author, Tel./fax: +86 532 83780377.

   E-mail address: [kychgt@qdu.edu.cn](mailto:kychgt@qdu.edu.cn) (G. Han). [↑](#footnote-ref-2)
